# Effects of timing of vitrectomy performed for open-globe injury patients on the thickness of retinal nerve fiber layer

**DOI:** 10.12669/pjms.311.6088

**Published:** 2015

**Authors:** Xiaoming Chen, Yanni Zhu, Shuqiong Hu, Yanhua Zhu

**Affiliations:** 1 Dr. Xiaoming Chen, Medical School of Yangtze University, Jingzhou 434000, Hubei Province, P. R. China.; 2 Dr. Yanni Zhu, Medical School of Yangtze University, Jingzhou 434000, Hubei Province, P. R. China.; 3 Dr. Shuqiong Hu, Medical School of Yangtze University, Jingzhou 434000, Hubei Province, P. R. China.; 4 Dr. Yanhua Zhu, Medical School of Yangtze University, Jingzhou 434000, Hubei Province, P. R. China.

**Keywords:** Traumatic optic neuropathy, Vitrectomy, Surgical timing, Retinal nerve fiber layer, Optical coherence tomography

## Abstract

**Objective:**

To study the effects of timing of vitrectomy performed for open-globe injury patients on the thickness of retinal nerve fiber layer (RNFL).

**Methods:**

A total of 120 patients with traumatic optic neuropathy (TON) were selected and divided into a treatment group and a control group by random draw (n=60). Vitrectomy was performed within one week upon injury for treatment group and after one week for control group. The thickness of RNFL was observed by optical coherence tomography.

**Results:**

All surgeries were conducted successfully, without severe complications. The best corrected visual acuity of treatment group surpassed that of control group one month after surgery, and treatment group had an obviously higher overall effective rate (95.0%) than control group did (81.7%). The incidence rate of postoperative complications in treatment group (6.7%) was significantly lower than that of control group (28.3%) (P<0.05). Logistic multivariate regression analysis showed that vitrectomy timing and postoperative complications were independent risk factors of prognosis (P<0.05). Both groups had significantly thinner RNFLs one week after surgery (P<0.05), and treatment group almost recovered within one month (P>0.05).

**Conclusion:**

Early vitrectomy effectively augmented the visual acuity of patients with TON, decreased complications, affected RNFL thickness reversibly, and improved prognosis.

## INTRODUCTION

Traumatic optic neuropathy (TON), as one of the main open-globe injuries, results from closed craniofacial injury. The patients with TON, in general, suffer from severe visual impairment or even loss of vision that can hardly be recovered naturally or by treatment, with poor outcomes and prognosis.^1^^,^^2^ With increasing traffic accidents due to economic development, TON occurs more frequently and thus has attracted wide attention.^3^ Currently, TON is mainly treated by early use of considerable glucocorticoids and vitrectomy, but there remain controversies over the timing, therapeutic effects and prognosis.^4^^,^^5^ It is now well-established that vitrectomy should be conducted as soon as possible to improve the therapeutic effects, but the specific timing has not been determined.^6^^,^^7^

Pathologically, TON is induced by the loss of retinal ganglion cells (RGCs) from the retinal nerve fiber layer (RNFL).^8^ Upon retinal optic nerve injury, RGCs gradually decrease and RNFL attenuates, thus reducing the visual acuity of patients. To this end, the aim of vitrectomy is to protect the survival RGCs after primary injury to recover visual acuity.^9^ Optical coherence tomography (OCT), which is a high-resolution tomographic imaging system, can directly measure the retinal thickness by imaging and clearly displaying the retinal tomographic structure. As a result, it is possible to better carry out biopsy.^10^^,^^11^

In this study, the thickness of RNFL was measured by OCT to analyze the influence of timing of vitrectomy performed for open-globe injury patients, aiming to postulate the disease development procedure and the optimum timing for surgery.

## METHODS

***Subjects: ***A total of 120 patients with traumatic optic neuropathy, who were treated in our hospital from September 2009 to November 2013, were selected in this study.

***Inclusion criteria:*** The patients conforming to the diagnostic standards of TON; the patients with unilateral eye injury (the other eye was healthy); the patients aged 18-60 years old; the patients with written consent.

***Exclusion criteria:*** The patients with incomplete eyeball, fundus hemorrhage, retinal edema and optic disc anomalies; the patients with glaucoma, retinal vascular disease, macular degeneration and retinal detachment that may alter the retinal optic nerve; the patients who had used drugs with ocular toxicity; the patients with refractive media opacity or fixed parallax that may interfere with the detection of OCT. There were 78 males and 42 females who were aged from 21 to 59 years old (average: 42.63 ± 4.15). Sixty-eight left eyes and fifty-two right eyes were included. Causes of injury: 60 cases of traffic accident injury, 32 cases of blast injury, 20 cases of blow injury, 6 cases of boxing injury, and 2 cases of foreign-body stabbing injury. Fifteen patients had the best corrected visual acuities lower than 0.01, and those of 65 cases and 40 cases were no less than 0.1 to lower than 0.3 and no less than 0.3 respectively. The patients were then divided into a treatment group and a control group by random draw (n=60), and the baseline data of the two groups were similar (P>0.05).

***Surgical methods: ***Vitrectomy was performed within one week upon injury for the treatment group and after one week for the control group. The patients were subjected to block anesthesia by injecting 2% lidocaine and 0.75% bupivacaine (v/v, 1/1) into the ciliary ganglion. Afterwards, the eyelid was opened and the conjunctival sac was rinsed by antibiotic solution. The bulbar conjunctiva was opened at the 2-o'clock, 7-o'clock and 10-o'clock positions, and the sclera was electrocoagulated. Then a closed incision was made 3.5 mm from the corneal limbus by a sclerotome, which was connected with an infusion tube at the 7-o'clock position. Subsequently, the vitreous body was resected and vitreous hemorrhage was cleared, and the retina was reset before infusing silicone oil. The scleral incision was thereafter interrupted-sutured to reset the position of the conjunctiva, and gentamicin in combination with dexamethasone was injected subconjunctivally. Finally, the treated eye was coated with ointment, and both eyes were covered.

Meanwhile, laser therapy was also performed, and the patients were administered antibiotics and hormones after surgery when necessary.

***Observation indices:*** *Eye examination:* The best corrected visual acuity was examined before surgery and one week, one month after respectively by using an international standard eye chart.

***Examination of RNFL:*** The thickness (μm) of RNFL was observed by ring-like OCT centered at the optic disc toward each axial direction with a diameter of 3.4 mm, and was measured by a computer. The measurement was completed by the same personnel under the same conditions.

***Observation of complications:*** Complications in the postoperative one month, including hypotony, hyphema, secondary glaucoma, choroidal detachment and uveal reaction, were observed.

***Determination of overall effective rate:*** Visual acuity was increased by three lines in the standard logarithmic visual acuity chart, with evidently improved clinical signs and mitigated visual field abnormality.

***Statistical analysis: ***All data were analyzed by SPSS 15.0. Inter-group comparisons were performed by Chi-square test, Wilcoxon rank sum test, t test and analysis of variance. P<0.05 was considered statistically significant. The relationships between the epidemiological state, disease state, surgical outcome and overall therapeutic effects and prognosis were evaluated by one-way univariate analysis.

## RESULTS

***Changes of visual acuity: ***One month after surgery, the best corrected visual acuity of the treatment group significantly excelled that of the control group (P<0.05) (Table-I).

***Overall effective rate: ***The treatment group had a significantly higher overall effective rate (95.0%) than the control group did (81.7%) in the postoperative 1st month (P<0.05) (Table-II).

***Postoperative complications: ***All the surgeries were conducted successfully, without any severe complications. The incidence of postoperative complications in the treatment group (6.7%) was significantly lower than that of the control group (28.3%) (P<0.05) (Table-III).

**Fig.1:**
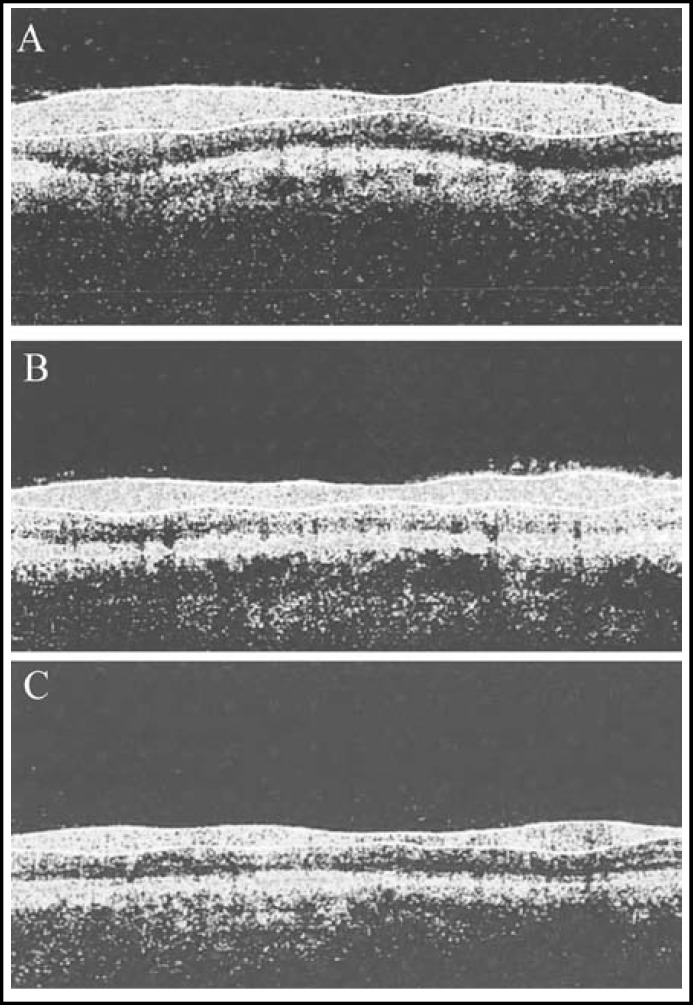
Preoperative and postoperative RNFL thicknesses of the treatment group. (A) Preoperative; (B) postoperative 1st week; (C) postoperative 1st month

**Fig.2:**
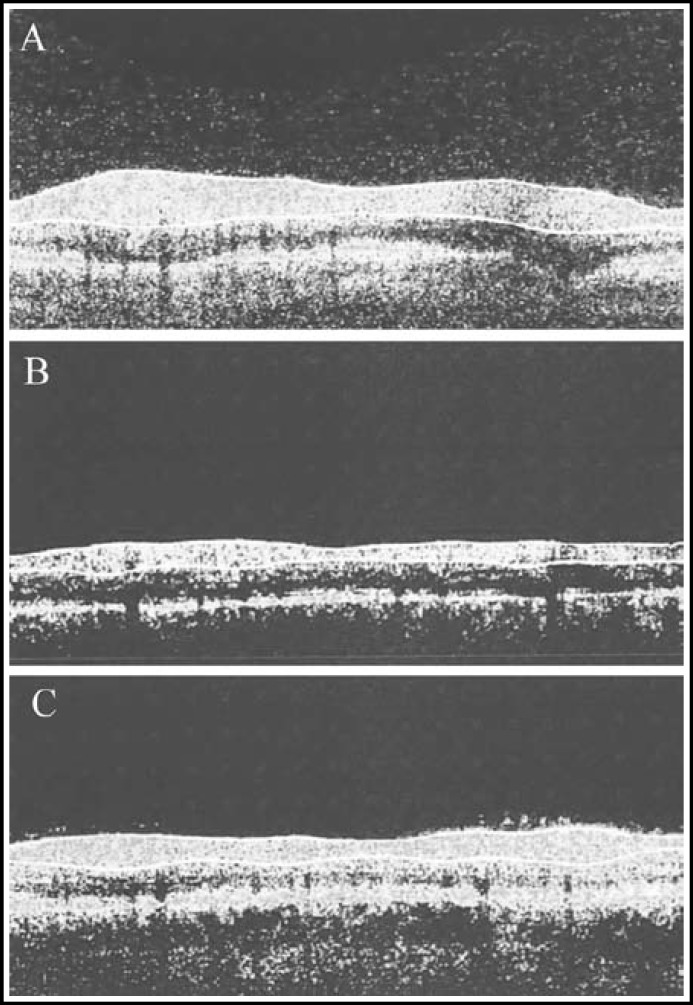
Preoperative and postoperative RNFL thicknesses of the control group. (A) Preoperative; (B) postoperative 1st week; (C) postoperative 1st month

***Changes of RNFL thickness: ***Both groups had significantly thinner retinal nerve fiber layers one week after surgery compared with those before surgery (P<0.05). However, only the thickness of the treatment group recovered one month after surgery (P>0.05) (Fig. 1), while that of the control group failed to do so (P<0.05) (Fig.2). Meanwhile, there was a significant difference between the two groups (P<0.05) (Table-IV).

***Analysis of prognostic factors: ***Logistic multivariate regression analysis showed that the time of vitrectomy and postoperative complications were the independent risk factors of prognostic effective rate (P<0.05) (Table-V).

## DISCUSSION

As the most important sense organ in human body, eye is subject to various injuries that endanger human health and even lead to disability or blindness. Eye injuries usually have poor prognosis owing to unexpected accidents and complicated symptoms.^12^ Of all open-globe injuries, TON is common among middle-aged and young men because of traffic and fall accidents. At present, the mechanism for TON remains unclear. Upon retinal optic nerve injury, the retina becomes incomplete due to axonal mechanical damages and undergoes secondary ganglion apoptosis and necrosis, eventually giving rise to irreversible visual decline and loss.^13^ In addition, there remains controversy over the treatment of TON, which is now mainly performed by surgery in combination with glucocorticoid administration.^14^

 Particularly, vitrectomy is given first priority to relieve edema-induced optic oppression, to improve local blood circulation, to increase blood flow within the optic nerve, and to eliminate secondary optic nerve injury.^15^ Nevertheless, vitrectomy may result in severe postoperative complications. It has been reported that early vitrectomy can remove blood clots and accelerate recovery. In case of proliferative changes, however, neovascular membrane or cord is commonly organized, thus requiring complex surgical protocols and decelerating vision recovery. Upon aggravation, visual acuity is bound to decrease owing to complicated retinal detachment.^16^ All the surgeries were conducted successfully, without any severe complications. The best corrected visual acuity of the treatment group was better than that of the control group one month after surgery, and the treatment group had a significantly higher overall effective rate (95.0%) than the control group did (81.7%) (P<0.05).

In the early stage of open-globe injury, bleeding and toxic substances do not induce macular edema or retinal traction by not destructing the macula lutea. Hence, it is highly recommended to perform vitrectomy as soon as possible.^17^ In the late stage, TON patients may suffer from secondary pathological changes that lead to the dysfunction and death of retinal ganglion cells, during which the macula lutea and the vitreous base are dragged by the vitreous body. As a result, the patients may succumb to postoperative complications because of retinal damages.^18^ The incidence of postoperative complications in the treatment group (6.7%) was significantly lower than that of the control group (28.3%) (P<0.05). Logistic multivariate regression analysis showed that the time of vitrectomy and postoperative complications were the independent risk factors of prognosis (P<0.05). Moreover, vitrectomy blocks the axonal transport of optic nerve fibers by pulling the fibers and the optic disc-nourishing blood vessels. Accordingly, it is necessary to perform vitrectomy as soon as possible to minimize the injuries to the frontal optic nerve.

**Table-I:** Postoperative best corrected visual acuities (n).

**Group**	**Case No.**		**<0.1**	**≥0.1＜0.3**	**≥0.3**
Treatment group	60		0 (0.0%)	15 (25.0%)	45 (75.0%)
Control group	60		5 (8.3%)	27 (45.0%)	28 (46.7%)
χ²			9.445		
P			<0.05		

**Table-II:** Postoperative overall effective rates (n).

**Group**	**Case No.**	**Effective**	**Ineffective**	**Overall effective rate**
Treatment group	60	57	3	95.0%
Control group	60	49	11	81.7%
χ²				4.874
P				<0.05

**Table-III:** Postoperative complications (n).

**Group**	**Case No.**	**Hypotony**	**Hyphema**	**Secondary glaucoma**	**Choroidal detachment**	**Uveal reaction**	**Total**
Treatment group	60	1	0	2	0	1	4 (6.7%)
Control group	60	2	2	6	2	5	17 (28.3%)
χ²							6.881
P							<0.05

**Table-IV:** Changes of RNFL thickness at different time points (μm, x±s).

**Group**	**Case number (n)**	**Before**	**Postoperative 1** ^st^ ** week**	**Postoperative 1** ^st^ ** month**
Treatment group	60	0.251±0.008	0.230±0.091	0.249±0.127
Control group	60	0.253±0.010	0.211±0.100	0.225±0.045
t		0.085	1.025	4.125
P		<0.05	<0.05	<0.05

**Table-V:** Logistic multivariate regression analysis of prognostic factors

**Factor**	**β**	**SE**	**P**	**OR**
Surgical time	2.482	1.062	0.002	0.095
Postoperative complications	2.239	1.003	0.012	0.108

RNFL of normal people is double hump-shaped, the thickness of which represents visual functions. First, decreased vision mainly results from the loss of retinal ganglion cells which can be reflected by the thickness changes of RNFL.^19^ Second, upon optic nerve injury, the preservation and recovery of vision are predominantly controlled by the number of survived retinal ganglion cells and the regeneration of axons. In other words, the thickness of RNFL suggests the changes of visual function.^20^ As a novel non-contacting, non-traumatic optical imaging technique, OCT is safe, accurate and highly repeatable, with high axial resolution that can directly, clearly display the topographic changes of retinochoroidal tissues.^21^ Both groups had significantly thinner retinal nerve fiber layers one week after surgery (P<0.05), and the thickness of the treatment group recovered one month after surgery (P>0.05). In the meantime, there was a significant inter-group difference (P<0.05). The outcomes herein suggested that early vitrectomy affected the morphology of RNFL, but the influences were reversible, without or only slightly affecting retinal functions.

In summary, TON is one of the main blinding eye diseases, which can be effectively mitigated by early vitrectomy that better augments visual acuity and reduces complications. Furthermore, the effects on the thickness of RNFL are reversible, thereby giving satisfactory prognosis.

## Authors Contribution:

**XMC** **&** **YNZ** conceived, designed and did statistical analysis & editing of manuscript.

**XMC** **,** **YNZ** **& SQH** did data collection and manuscript writing.

**YHZ** did review and final approval of manuscript.

**YHZ** takes the responsibility and is accountable for all aspects of the work in ensuring that questions related to the accuracy or integrity of any part of the work are appropriately investigated and resolved.
